# The Roles of MicroRNAs in Breast Cancer

**DOI:** 10.3390/cancers7020598

**Published:** 2015-04-09

**Authors:** Ryou-u Takahashi, Hiroaki Miyazaki, Takahiro Ochiya

**Affiliations:** 1 Division of Molecular and Cellular Medicine, National Cancer Center Research Institute 1-1, Tsukiji 5-chome, Chuo-ku, Tokyo 104-0045, Japan; E-Mails: rytakaha@ncc.go.jp (R.T.); himiyaza@ncc.go.jp (H.M.); 2 Department of Oral and Maxillofacial Surgery, Showa University School of Dentistry, 1-5-8 Hatanodai Shinagawa-ku, Tokyo 142-8555, Japan

**Keywords:** microRNA, therapy, diagnosis, breast cancer

## Abstract

MicroRNAs (miRNAs) constitute a large family of small, approximately 20–22 nucleotide, non-coding RNAs that regulate the expression of target genes, mainly at the post-transcriptional level. Accumulating lines of evidence have indicated that miRNAs play important roles in the maintenance of biological homeostasis and that aberrant expression levels of miRNAs are associated with the onset of many diseases, including cancer. In various cancers, miRNAs play important roles in tumor initiation, drug resistance and metastasis. Recent studies reported that miRNAs could also be secreted via small endosome-derived vesicles called exosomes, which are derived from multiple cell types, including dendritic cells, lymphocytes, and tumor cells. Exosomal miRNAs play an important role in cell-to-cell communication and have been investigated as prognostic and diagnostic biomarkers. In this review, we summarize the major findings related to the functions of miRNAs in breast cancer, which is the most frequent cancer in women, and discuss the potential clinical uses of miRNAs, including their roles as therapeutic targets and diagnostic markers.

## 1. Introduction

According to the International Agency for Research on Cancer (http://www.iarc.fr/), in 2012, 1.7 million women (11.9%) were diagnosed with breast cancer and 6.3 million women were alive who had been diagnosed with breast cancer in the past five years. Breast cancer is still the most common cause of cancer death among women, accounting for 522,000 deaths in 2012 [1]. Breast tumors are very heterogeneous and can be classified into several subtypes based on distinct gene expression profiles [2]. To develop more effective treatments, it is essential to understand the molecular mechanisms involved in breast tumor development and the acquisition of malignancy. In addition, translational research, which is based on basic cancer research, is required to overcome intractable cancers, such as therapy-resistant and metastatic cancers.

It is well established that microRNAs (miRNAs) are critical regulators of global mRNA expression in both normal and abnormal biological processes, including cancer. Dysregulation of miRNAs occurs in various types of cancers and is associated with tumor initiation, drug resistance, and metastasis; therefore, therapeutic strategies based on modulating the expression levels of miRNAs and identifying their targets are promising approaches for cancer treatment. Recent studies reported that miRNAs are secreted from exosomes and are present in various body fluids. Interestingly, the amounts of circulating miRNAs differ between cancer patients and healthy donors [3]. In this review, we summarize the major functions of miRNAs in breast tumor development and discuss the clinical applications of modulating miRNA expression and detecting circulating levels of miRNAs.

## 2. Biosynthesis and Functions of miRNAs

**Figure 1:**
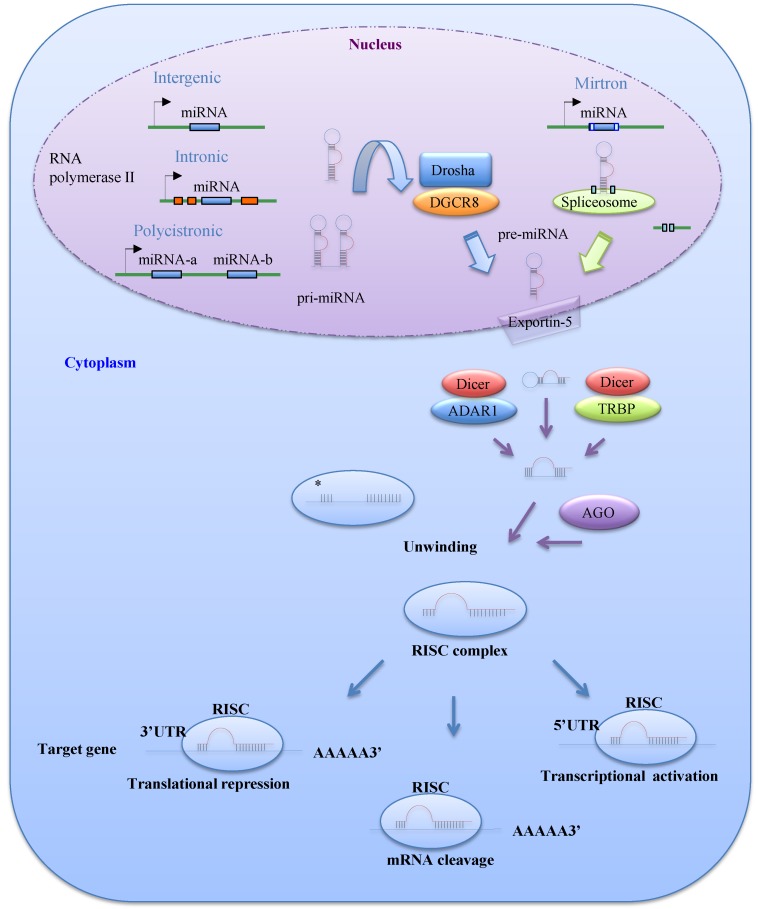
The biogenesis and function of microRNAs (miRNAs). MiRNAs are initially transcribed by RNA polymerase II or III as pri-miRNAs, which are processed into pre-miRNAs in the nucleus by Drosha-DGCR8. Precursor miRNAs (pre-miRNAs) can also be generated via the mirtron pathway. The products of pri-miRNA cleavage, the pre-miRNAs, are exported to the cytoplasm through exportin-5 and then cleaved in a complex comprising Dicer and transactivating response RNA-binding protein (TRBP) or adenosine deaminase acting on RNA 1 (ADAR1). The functional strand of a mature miRNA is incorporated into the RNA-induced silencing complex (RISC), which contains GW182 and AGO proteins. As a part of this complex, the mature miRNA modulates gene expression by binding to partially complementary sequences in the 3'UTRs or 5'UTRs of target mRNAs, leading to mRNA degradation, translational inhibition, or transcriptional activation.

MiRNAs are 21–25 nucleotide non-coding RNAs that regulate gene expression at the post-transcriptional level. MiRNAs are transcribed mainly by RNA polymerase II as long primary transcripts called pri-miRNAs, which are characterized by hairpin structures. In the nucleus, these pri-miRNAs are processed into 70–100 nucleotide precursor miRNAs (pre-miRNAs) by Drosha, an RNase III enzyme, and its co-factor DGCR8 (Figure 1). The *DGCR8* gene is located at chromosomal region 22q11.2 and its heterozygous deletion results in DiGeorge syndrome, the most common human genetic deletion syndrome [4,5]. Several pre-miRNAs are also produced by the mirtron pathway, in which introns are spliced and debranched by lariat debranching enzyme (Figure 1) [6,7]. Pre-miRNAs are exported to the cytoplasm by Exportin-5, which is a member of the Ran-dependent nuclear transport receptor family [8], and then cleaved into miRNA:miRNA* duplexes by a complex comprising the RNase III enzyme Dicer and transactivating response RNA-binding protein (TRBP). A recent study demonstrated that adenosine deaminase acting on RNA 1, which is involved in RNA editing, can also form a protein complex with Dicer in place of TRBP and promote miRNA processing [9].

One of the two miRNA strands in the duplex is selected as a guide strand, whereas the complementary strand (miRNA*) is usually degraded [10]. MiRNA* strands were originally thought to be non-functional; however, recent evidence suggests that they may play significant biological roles [11]. Mature miRNAs are incorporated into the RNA-induced silencing complex, which contains the GW182 and Argonaute (AGO) proteins. As a part of this complex, mature miRNAs play a role in gene regulation by interacting with partially complementary sequences in the 3'UTRs or miRNA response elements of target genes, leading to mRNA degradation or translation inhibition (Figure 1) [10]. Several studies have reported that miRNAs can also bind to the 5'UTR or open reading frame of their targets [12,13]. Liu *et al*. [12] reported that miR-483-5p, which is embedded in the insulin-like growth factor 2 (*IGF2*) gene, binds directly to the 5'UTR of this gene and induces its transcription in human fetal kidney and Wilms’ tumors.

## 3. miRNAs in Cancer

Accumulating lines of evidence have shown that (i) miRNAs play important roles in tumor development; (ii) miRNAs are differentially expressed depending on the molecular subtypes of tumors, and (iii) the expression profiles of individual miRNAs classify tumor malignancies [14,15]. Amplification of chromosomal regions encoding oncogenic miRNAs that inhibit tumor suppressor genes is also associated with cancer development; amplification of these regions leads to the up-regulation of oncogenic miRNAs and subsequent silencing of tumor suppressor genes [16]. On the other hand, tumor-suppressive miRNAs are often located in chromosomal fragile sites; deletion or mutation of these regions results in a reduction or loss of expression of tumor-suppressive miRNAs and subsequent up-regulation of their target oncogenes [17].

Dysregulation of miRNA expression affects the processes associated with cancer progression, such as the escape from apoptosis, tissue invasion, and metastasis [18,19]. Recent studies have suggested that miRNAs are involved in tumor initiation through the regulation of cancer stem cell (CSC) properties, including self-renewal ability, tumorigenicity and drug resistance [20,21,22]. In breast cancer, a number of miRNAs have been identified as tumor suppressors or oncogenes and have been characterized as critical regulators of tumor initiation, metastasis and chemoresistance (Table 1).

**Table 1:** The functions of miRNAs in breast cancer.

Phenotype		miRNA	Target Genes	References
Tumor initiation	Inhibition of self-renewal activity and de-differentiation	let-7	*RAS*, *HMGA2*	[23]
		miR-200c	*BMI-1*	[21]
	EMT	miR-200 family & miR-205	*ZEB1*	[24]
		miR-103/107	*DICER*	[25]
		miR-22	TET family (*TET1-3*)	[26]
Drug resistance		miR-451	*ABCB1*	[27]
		miR-326	*ABCC1*	[28]
		miR-487a	*ABCG2*	[29]
		miR-221/222	*p27^kip1^*	[30]
		miR-30c	*TWF1* and *IL-11*	[31]
		miR-31	*PKCepsilon*	[32]
Invasion and metastasis		miR-10b	*HOXD1*	[33]
		miR-335	*SOX4*, *TNC*	[34]
		miR-31	*RhoA & ITGA5*	[35]
		miR-34	*Snail*	[36]
		miR-29b	*VEGFA, ANGPTL4, LOX*	[37]
		miR-708	*NNAT*	[38]

Abbreviations: ANGPTL4, angiopoietin-like 4; BMI-1, B cell-specific Moloney murine leukemia virus integration site 1; EMT, epithelial to mesenchymal transition; HOXD1, Homeobox D1; IL-11, interleukin-11; ITGA5, integrin 5α; LOX, lysyl oxidase; PKCepsilon, protein kinase C epsilon; TNC, tenascin C; TWF1, twinfilin 1; VEGFA, vascular endothelial growth factor A.

## 4. The Roles of miRNAs in Breast Cancer Development

### 4.1. Tumor Initiation

A number of studies have suggested that cancer-initiating cells or CSCs are responsible for tumor development and progression. These cells share a variety of biological properties with normal somatic stem cells, including the capacity for asymmetric cell division and the ability to efflux small compunds [39]; however, CSCs differ from normal stem cells in their tumor seeding and metastatic abilities. In addition, the phenotypic plasticity of CSCs, which is their ability to differentiate into non-CSCs, is thought to be an important factor for preventing tumor malignancy [40]. Therefore, the CSC theory of cancer development is generally accepted in the fields of basic and translational cancer research.

The first CSCs in solid tumors were identified and isolated from breast cancers. In 2003, Al-Hajj *et al.* [41] reported that CD44^+^/CD24^−/low^ Lineage^−^ cells from human breast specimens show a remarkably high tumor-seeding ability, and in 2007, Yu *et al*. [23] reported that let-7 is a master regulator of CSC properties such as self-renewal activity and tumor-seeding ability. Using mammosphere culture conditions and treatment with anti-cancer reagents, Yu *et al*. confirmed that CSCs show a CD44^+^/CD24^−/low^ antigen phenotype and have significant down-regulation of let-7 expression; furthermore, they also demonstrated that let-7 inhibits the self-renewal and de-differentiation of breast cancer cells via direct targeting of the genes encoding RAS and high mobility group AT-hook 2 (HMGA2), respectively.

In 2008, Mani *et al*. [42] reported that CD44^+^/CD24^−/low^ cell populations from cancerous breast tissues show the features of epithelial to mesenchymal transition (EMT) and high tumorigenicity. Because EMT is often observed during tumor invasion and metastasis, the genetic controls and molecular mechanisms underlying the acquisition of invasiveness and the subsequent systemic spread of metastatic cells have been areas of intensive research. The EMT phenotype is characterized by the loss of epithelial markers such as E-cadherin, the up-regulation of mesenchymal markers such as N-cadherin and vimentin, the loss of cell-cell adhesion and cell polarity, and the acquisition of cell invasive capabilities. A molecular link between EMT and miRNAs was reported by Gregory *et al*. [24], who found that miR-205 and five members of the miR-200 family, namely, miR-200a, miR-200b, miR-200c, miR-141, and miR-429, are selectively downregulated in Madin Darby canine kidney cells undergoing EMT. The miR-200 family is classified into two clusters, namely, miR-200a, miR-200b, and miR-429 on human chromosome 1, and miR-200c and miR-141 on human chromosome 12 [24]. Expression of the miR-200 family inhibits the EMT phenotype induced by transforming growth factor-β via direct targeting of the genes encoding the E-cadherin transcriptional repressors zinc finger E-box-binding homeobox 1 (ZEB1) and ZEB2 [24]. On the other hand, ZEB1 suppresses the transcription of miR-141 and miR-200c, which are strong inducers of epithelial differentiation. Therefore, the EMT phenotype is tightly regulated by a reciprocal interaction between the miR-200 family and ZEB1 [43].

The EMT phenotype is also induced by miR-103/107 in breast cancer cells. Martello *et al*. [25] found that miR-103/107 attenuates miRNA biosynthesis by targeting the gene encoding Dicer, leading to global down-regulation of miRNAs, including the miR-200 family, and the subsequent development of EMT and a metastatic phenotype of epithelial cancer cells. Recently, Song *et al*. [26] reported that the expression levels of the miR-200 family are epigenetically regulated via miR-22-mediated suppression of ten eleven translocation (TET) family members (TET1-3), which induce DNA demethylation by converting 5-methyl-cytosine to 5-hydroxymethylcytosine. Because the TET family is involved in demethylation of the miR-200 promoter, miR-22 promotes the acquisition of CSC properties, such as EMT and a metastatic phenotype via suppression of the miR-200 family. Song *et al*. provided the first evidence that chromatin-remodeling systems with opposing effects on cell fate (self-renewal *versus* differentiation) and EMT induction are regulated by the balance of opposing sets of miRNAs.

### 4.2. Drug Resistance

The resistance to chemotherapy and molecularly targeted drugs is a serious problem facing current cancer research [44]. The mechanisms underlying the acquistion of drug resistance are classified broadly into two categories, namely, the alteration of drug transporters that efflux anti-cancer agents [45,46], and the activation of anti-apoptotic and cell-survival pathways [32]. Several miRNAs have been identified as critical regulators of drug resistance in breast cancer (Table 1). The differential expression profiles of miRNAs in responders and non-responders to chemotherapy are frequently used to identify drug resistance-related miRNAs and their targets [31,47].

One of the major causes of drug resistance is the elevated expression of ATP-binding cassette (ABC) transporters [48,49,50]. ABC transporters efflux small molecules, including anti-cancer agents, and 49 members of this protein family have been identified to date [44]. The roles of three ABC transporters (ABCB1, ABCG2 and ABCC1) in multi-drug resistance associated with the efflux of various hydrophobic compounds, including major anti-cancer agents, such as taxanes, anthracyclines and anti-metabolites, have been studied in some detail [44,51]. ABCB1, the first ABC transporter to be identified [52], is reportedly associated with the failure of chemotherapy treatment of various cancers, including breast, colon and lung cancers [45,53,54]. ABCC1, also known as MRP1, is associated with chemotherapy resistance of prostate, breast, and lung cancers [55,56,57], and ABCG2, which is also known as BCRP, is involved in the chemoresistance of breast cancer and leukemia [50,58]. Notably, recent studies reported that CSCs from various types of cancers have elevated expression levels of these drug efflux proteins [23,59]. Several studies have demonstrated that miRNAs are involved in the post-transcriptional regulation of the genes encoding ABC family proteins [27,28,29]. In breast cancer, miR-451 and miR-326 increase the chemosensitivity of cells to doxorubicin via direct targeting of *ABCB1* and *ABCC1*, respectively [27,28]. Similarly, miR-487a regulates the chemosensitivity of breast cancer cells to mitoxantrone via direct targeting of *ABCG2* [29].

Recent studies revealed that miRNAs are also associated with another mechanism of drug resistance [30,31,60]. Miller *et al*. [30] reported elevated expression levels of miR-221/222 in tamoxifen-resistant luminal-type breast cancer cells. Because miR-221/222 is a negative regulator of p27^kip1^, a cell-cycle inhibitor and tumor suppressor [60], and tamoxifen-resistant breast cancer cells display up-regulation of these miRNAs and significant reductions in p27^kip1^ levels [30], miR-221/222 is thought to regulate tamoxifen sensitivy via direct targeting of *p27^kip1^*. Moreover, Pichiorri *et al.* [61] found that miR-221/222 expression is specifically modulated at the post-transcriptional level by nucleolin and demonstrated that targeting nucleolin effectively suppresses breast tumor malignancy. Bockhorn *et al*. [31] reported that miR-30c suppresses interleukin-11 expression and inhibits the resistance of breast cancers to paclitaxel and doxorubicin via direct targeting of the actin-binding protein twinfilin 1, which promotes the EMT phenotype.

### 4.3. Metastasis

Metastasis, which is a multi-step process involved in cancer aggressiveness, is the leading cause of cancer deaths [62]; therefore, it is important to elucidate the mechanisms regulating this process. Metastasis is caused by multiple intricate steps that arise from the primary tumor site [63]. The main step of metastasis is initiated from extensive vascularization at the primary tumor site; the cells then show a loss of adhesion and acquire an invasive phenotype, leading to their subsequent detachment and mobilization. The EMT process is thought to be important for the mobilization step. After circulation in blood vessels, the tumor cells enter new tissues and colonize at distant sites.

In addition to regulating chemoresistance, miRNAs are also involved in the modulation of metastatic processes (Table 1). MiR-10 was first identified as a key regulator of breast cancer metastasis; Ma *et al*. showed that the expression levels of miR-10b are higher in metastatic than non-metastatic breast cancer cell lines [33]. In non-metastatic breast cancer cell lines, ectopic expression of miR-10b induces the up-regulation of RAS homolog gene family member C via direct targeting of the gene encoding homeobox D10, resulting in the promotion of invasion and metastasis [33]. By contrast, Tavazoie *et al.* identified miR-126, miR-206 and miR-335 as suppressors of breast cancer metastasis [34]; restoration of miR-126 reduces cell proliferation and tumor growth, while miR-206 and miR-335 inhibit metastatic cell invasion. MiR-335 exerts its inhibitory effects on breast cancer metastasis through direct suppression of the genes encoding the SOX4 transcription factor and the extracellular matrix component tenascin C. In addition, Valastyan *et al*. [35] reported that miR-31 is downregulated in metastatic breast cancer cells and inhibits the invasiveness and metastasis of these cells via direct targeting of multiple metastasis-associated genes, such as *RhoA* and *ITGA5* (encoding integrin 5α).

The EMT program is often activated during tumor invasion and metastasis; hence, the genetic changes and molecular mechanisms by which cancer cells acquire invasiveness and their subsequent metastatic ability have been areas of intensive research. Kim *et al.* reported that miR-34a, which is a transcriptional target of p53 [64], inhibits the invasiveness of breast cancer cells via repression of EMT and the zinc finger transcriptional repressor Snail [36]. As described above, the balance between the expression levels of miR-22 and the miR-200 family also regulates the EMT phenotype in breast cancer [24,26]; hence, these miRNAs are thought to be important factors that control the initial step of metastasis. In addition, Chou *et al*. [37] demonstrated that the transcription factor GATA3 suppresses breast tumor metastasis by up-regulating miR-29b. GATA3 is required for the maintenance of luminal epithelial cell differentiation in the mammary gland and loss of miR-29b is correlated with poor prognosis in breast cancer patients [65,66,67]. MiR-29b inhibits breast cancer metastasis by suppressing a network of pro-metastatic regulators associated with angiogenesis, collagen remodeling and proteolysis [37] (Table 1). Ryu *et al.* [38] reported that miR-708 inhibits breast cancer cell migration and metastasis by targeting the endoplasmic reticulum protein neuronatin, which regulates the intracellular Ca^2+^ level. Suppression of the gene encoding neuronatin (*NNAT*) by miR-708 causes aberrant Ca^2+^ regulation, resulting in inactivation of cell migration-associated proteins such as extracellular signal-regulated kinase and focal adhesion kinase [38,68]. Finally, Shen *et al*. [69] reported that in hypoxic conditions, epidermal growth factor receptor inhibits the physical interaction between AGO2 and Dicer through phosphorylation of AGO2 at Tyr393, resulting in inhibition of the processing of tumor suppressor-like pre-miRNAs to mature miRNAs. Shen *et al.* also found that the phosphorylation of AGO2 at Tyr393 is associated with poor prognosis in breast cancer patients [69].

### 4.4. Secreted miRNAs

#### 4.4.1. Exosomes

In 1979, Taylor *et al.* [70] were the first to report that several types of tumor cells release or shed intact microvesicles composed of membrane proteins. Subsequently, microvesicles derived from various types of cells have been detected in biologic fluids, such as blood, urine, breast milk, and saliva; such vesicles are increasingly recognized as important tools for intercellular and extracellular communications [71,72,73]. Depending on their size, markers, cargoes, and function, microvesicles have also been defined as apoptotic bodies, extracellular vesicles and exosomes [74]. Exosomes are defined as 50–100 nm membrane vesicles derived from various types of cells in both physiological and pathological conditions [74]. One of the most exciting findings of a recent study by Valadi *et al*. [75] was that miRNAs packaged in exosomes, which can also harbor proteins, DNA, and mRNA, can be functionally transferred into target cells. In cancer biology, multiple lines of evidence have revealed that the components secreted by exosomes from cancer cells are associated with tumor development and malignancy [76,77,78]; therefore, many researchers are currently trying to remove or antagonize tumor-derived exosomes as a novel cancer therapy [79]. In addition, considering the potential of exosomes for cancer diagnosis, the development of devices that are capable of sensing small numbers of exosomes and profiling their contents is required, but is somewhat challenging.

#### 4.4.2. The Roles of Exosomal miRNAs in Breast Cancer Development

Exosomal miRNAs have been implicated in various aspects of breast tumor development [76,80,81]. In bone marrow metastasis of breast cancer, recurrence decades after the initial diagnosis and treatment implicates the long-term suvival of cancer cells in a dormant state. Lim *et al*. [80] showed that exosomal miRNAs derived from bone marrow stroma (miR-127, miR-197, miR-222, and miR-223) inhibit breast cancer cell proliferation via direct targeting of the *CXCL12* chemokine gene*,* leading to the induction or maintenace of a dormant state of breast cancer cells. On the other hand, exosomes from tumor-associated macrophages promote breast cancer invasion and metastasis. Yang *et al*. [81] reported that tumor-associated macrophages activated by interleukin-4 released from CD4^+^ T-cells transfer miR-223 to breast cancer cells via exosomes, and miR-223 induces the nuclear accumulation of β-catenin through direct targeting of myocyte enhancer factor 2C, resulting in the acquisition of invasiveness.

A recent study demonstrated that exosome-derived miRNAs promote the metastasis of breast cancer; Zhou *et al*. [76] reported that high expression levels and amounts of secreted miR-105 are associated with highly metastatic breast cancer cells. Cancer-secreted miR-105 disrupts tight junctions by suppressing the expression of the tight junction protein ZO-1 in distant organs. Therefore, miR-105-overexpressing breast cancer cells induce vascular permeability and show highly metastatic activity.

Exosomal miRNAs are also associated with the acquisition of drug resistance. Chen *et al*. [82] showed that exosomal miRNAs released from drug-resistant breast cancer cells reduce the chemosensitivity of drug-sensitive cancer cells; among these exosomal miRNAs (miR-17, miR-30a, miR-100, and miR-222), miR-222 is transferred to recipient cells and suppresses the expression of phosphatase and tensin homolog, which is involved in the resistance to adriamycin and docetaxel [83].

## 5. miRNA-Based Therapeutic Approaches to Breast Cancer Treatment

### 5.1. Therapeutic Modulation of miRNA Expression

Since miRNAs play important roles in the regulation of tumor initiation and development, modulation of miRNA activity is a promising approach for cancer treatment. Currently, many researchers are attempting to restore the function of tumor suppressor miRNAs using miRNA mimics or expression vectors, or inhibit the function of oncogenic miRNAs using antisense oligonucleotides or expression vectors containing complementary sequences [84,85].

### 5.2. Modulation of miRNA Activity

Improving the stability and tissue specific delivery of miRNA mimics is essential for the therapeutic modulation of miRNA activity. One strategy to stabilize miRNA mimics is chemical modification. Noguchi *et al*. [86] reported that modification of the passenger sequence and the addition of an aromatic benzene-pyridine analog to the 3'-overhang region of the double-stranded miR-205 promotes its resistance to nuclease degradation and stability *in vivo*. Takahashi *et al*. [87] also reported that the 2'-O-methyl-4'-thiol modification protects small RNAs from nuclease degradation and improves the binding affinity for their target sequence. Recently, miR-205 was identified as a critical regulator of mammary stem cell maintenace that acts by direct targeting of *ZEB1* and *NOTCH2* [24,88]; therefore, chemical modification of miR-205 might be beneficial to breast cancer treatment.

### 5.3. Targeted Delivery of miRNAs

For the clinical use of miRNAs, it is essential to consider that a single miRNA can regulate the expression of multiple genes. To minimize the repression of genes at non-disease sites, it is important to develop methods or techniques that enable the specific delivery of miRNAs to the intended site. Huang *et al*. [89] improved the delivery efficacy of miR-29b to leukemia cells using anionic lipopolyplex nanoparticles conjugated with transferrin. MiR-29b is downregulated in acute myeloid leukemia and up-regulation of miR-29b target genes contributes to myeloid leukemogenesis [90]; hence, targeted delivery of miR-29b using transferrin-conjugated nanoparticles improves its antileukemic activity both *in vitro* and *in vivo* [90].

Esposito *et al*. [91] developed a method for the specific delivery of miRNAs using nucleic acid aptamers. Using an aptamer that binds specifically to the receptor tyrosine kinase oncogene Axl [92], conjugated let-7g, which targets *HMGA2*, was selectively delivered to Axl-positive lung tumors and showed effective inhibition of tumor growth.

In breast cancer, let-7 suppresses breast cell self-renewal and tumorigenicity by targeting *H-RAS* and *HMGA2* [23], and miR-29b expression is regulatd by GATA3, which is a master controller of luminal differentiation that inhibits the acquisition of the EMT phenotype and metastasis [37]. Therefore, combined with specific aptamers or antibodies against breast CSC markers, the promising methods described by Huang *et al*. and Esposito *et al*. [89,91] might be useful approaches to improving the treatment of breast cancer patients. Although considerable efforts have been made to improve the target-binding affinities and nuclease resistances of the miRNA delivery systems described above, more research is required to improve the design of vehicles and methods for their specific delivery *in vivo*.

### 5.4. Exosome-Based Strategies for Cancer Diagnosis

Like gene expression profiles [2], miRNA expression profiles are also correlated with breast tumor development, progression and prognosis [93,94,95]. Because the amount and contents, including miRNAs, of exosomes reflect the pathological conditions of cancer patients [3], many researchers are trying to characterize and classify exosomes according to tumor malignancies.

Taylor *et al*. [3] were the first to report that the expression pattern of exosomal miRNAs is associated with tumor malignancy; while epithelial cell adhesion molecule-positive exosomes were detected in serum derived from ovarian benign disease and cancer, the exosomal miRNA profiles differed between cancer and benign patients. Zhu *et al.* [96] were the first to investigate the miRNA profiles in sera derived from cancer patients and healthy subjects; this group found that the serum levels of miR-155 differ between hormone-sensitive and -insensitive breast cancer patients. Furthermore, Wang *et al*. [97] performed a meta-analysis and found that the circulating level of miR-155 has potential value in the diagnosis of breast cancer. Recently, Dinami *et al*. [98] reported that elevated expression of miR-155 promotes telomere fragility and changes the structure of metaphase chromosomes via direct targeting of the shelterin component TERF1, and showed that miR-155 expression is associated with poor prognosis of luminal-type breast cancer patients. Schooneveld *et al*. [99] also found that the serum levels of several miRNAs differ between healthy subjects and metastatic breast cancer patients; compared with those in healthy subjects, the levels of miR-215, miR-299, and miR-411 were significantly lower in untreated patients with metastatic breast cancer. More recently, two cohort studies reported that the levels of some specific miRNAs in serum are associated with breast tumor subtype and stage [100,101]. Taken together, these findings indicate that serum-derived miRNAs, including exosomal miRNAs, might be surrogate biomarkers of the tumor stage and response to cancer therapy.

Recently, some groups succeeded in developing devices to detect and monitor microvesicles in clinical samples. Im *et al*. [102] developed a surface plasmon resonance-based assay system that uses optical transmission to detect label-free exosomal proteins. Using this system, CD24 and epithelial cell adhesion molecule were identified as specific antigens for tumor-derived exosomes in ovarian cancer [102]. Yoshioka *et al.* [103] also established a sensitive and rapid analytical technique for detecting and characterizing microvesicles in blood samples (at least 5 μL) from colon cancer patients. These reports suggest that exosomes may be promising candidate biomarkers for the early detection of cancers and for monitoring therapy response and the patient’s condition; however, more work is required to improve the sensitivities and specificities of detection systems for circulating miRNAs and exosomes.

## 6. Conclusions

In this review, we have summarized the roles of miRNAs in cancer biology, with a particular focus on breast cancer. Accumlating lines of evidence have revealed that abberant expression levels of miRNAs in both tumor tissues and body fluids are associated with the patient condition and tumor stage. In addition, breast cancer comprises several subtypes with different molecular profiles and biological phenotypes, and specific miRNAs are involved in the determination and regulation of such subtypes. Understanding the molecular mechanisms involved in miRNA expression and secretion, and profiling miRNA expression in different tissues and body fluids, are important topics in both basic and clinical research.
